# A successful management of left-sided posterior congenital diaphragmatic hernia of the jejunum, ileum, colon and left kidney: a case report

**DOI:** 10.1093/jscr/rjac521

**Published:** 2022-11-19

**Authors:** Zainab Al-Abdullah, Ruthwik Duvuru, Faisal A Nawaz, Farah Ennab, Temaa Alklani, Aftab Ahmed

**Affiliations:** College of Medicine, Mohammed Bin Rashid University of Medicine and Health Sciences, 505055 Dubai, United Arab Emirates; College of Medicine, Mohammed Bin Rashid University of Medicine and Health Sciences, 505055 Dubai, United Arab Emirates; College of Medicine, Mohammed Bin Rashid University of Medicine and Health Sciences, 505055 Dubai, United Arab Emirates; College of Medicine, Mohammed Bin Rashid University of Medicine and Health Sciences, 505055 Dubai, United Arab Emirates; Faculty of Medicine, Damascus University, Damascus, Syrian Arab Republic; Department of Pediatric Surgery, Mediclinic Welcare Hospital, Dubai, United Arab Emirates

## Abstract

Congenital diaphragmatic hernia (CDH) is a rare developmental anomaly in which abdominal contents herniate into the thoracic cavity due to underdevelopment of the diaphragm, possibly leading to pulmonary hypoplasia. Whereas surgery is not the first priority in treatment, it must be performed within a window of 2 weeks and after hemodynamic stability has been achieved. The patient described in this case report had a CDH of the jejunum, ileum, colon and left kidney diagnosed in a boy of South Asian origin who presented with tachypnea in the third hour of life. Imaging studies conducted included chest X-ray, chest ultrasound including echocardiogram, and abdominal and pelvic ultrasound. Treatment and management were successful despite complications. Future research on CDH is warranted in the populations in the Middle East, and local guidelines must be generated in order to improve diagnosis, treatment and prognosis.

## INTRODUCTION

Congenital diaphragmatic hernia (CDH) is a rare developmental anomaly in which abdominal contents herniate into the thoracic cavity through an underdeveloped diaphragm, possibly leading to pulmonary hypoplasia [1]. Significant risk factors include male sex and maternal age over 35 years [2, 3].

Counterintuitively, the treatment is aggressive pulmonary support, gentle ventilation and treatment of pulmonary hypertension [4, 5]. Whereas surgery is not the first-line treatment, it must be performed within a window of 2 weeks [4]. Imaging must include an echocardiogram to assess cardiac function and pulmonary vascular resistance [4, 6].

## CASE REPORT

The patient is a male of South Asian origin who was born at term with a CDH diagnosed hours after birth. He was a singleton pregnancy delivered through a lower segment C-section. The patient’s mother was in her early 30s, gravida 2 para 1. The mother had no active history of hypertension or diabetes during the pregnancy.

At birth, the patient’s APGAR score was noted to be 9 out of 10. Oxygen saturation was 95%, and the heart rate was 152 beats per minute. He weighed 3500 g, was 52 cm long and had a head circumference of 36 cm. The patient was comfortable with feeding and received the bacille Calmette–Guérin and hepatitis B vaccines.

In the third hour of life, the patient was tachypneic. Oxygen saturation was at 90%, and the chest was retracting during inspiration. Inhaled oxygen and empirical antibiotics were started, a septic screen was done and the patient was transferred to the neonatal intensive care unit.

**Figure 1:**
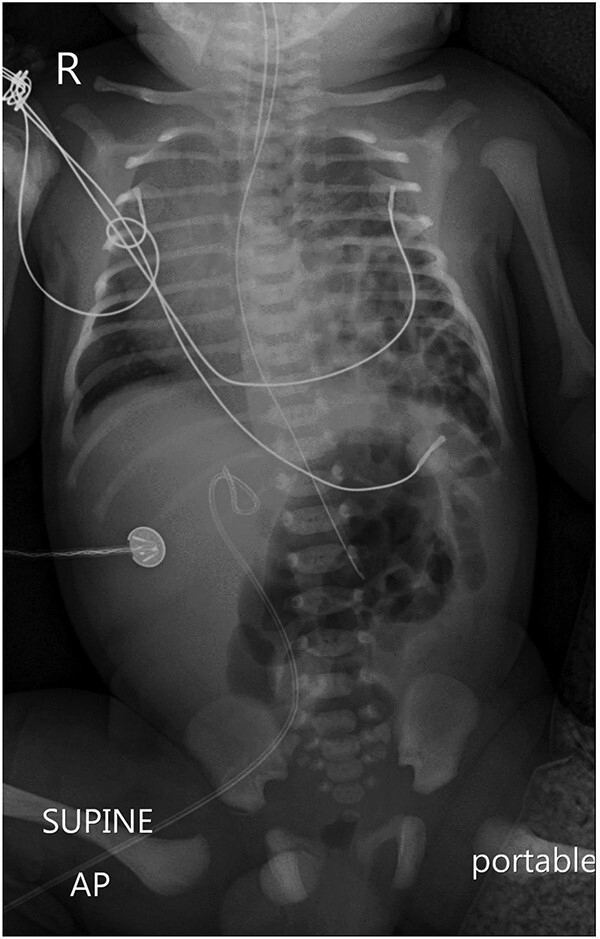
A portable X-ray photo of the chest, abdomen and pelvis shows the right mediastinal shift, total collapse of the left lung, partial collapse of the right lung and air-filled structures (bowel loops) extending from the abdomen into the chest.

A chest X-ray revealed a left-sided diaphragmatic herniation of bowel loops into the left hemithorax along with displacement of the cardiac shadow to the right (Fig. 1). Nasogastric and endotracheal tubes were also inserted. Umbilical arterial and venous catheter insertion was attempted but failed and removed the next day.

An echocardiogram showed cardiac and great vessel right shift. There was no sign of persistent pulmonary hypertension of the newborn. However, a small patent foreman ovale and patent ductus arteriosus with a restrictive left-to-right shunt were noted.

Initial abdominal and pelvic ultrasound did not visualise the left kidney. The herniated bowel had intrinsic peristalsis. The liver, gallbladder, spleen, stomach, right kidney and bladder were visualised normally.

On the second day of life, a 2-h-long corrective surgery relocated the patient’s ileum, jejunum, colon and left kidney—the latter was discovered to be dislocated during the surgery under general anesthesia. This was an open surgery whereby the incision was through the left subcostal space. A diaphragmatic defect was seen through which the ileum jejunum and colon were seen to pass into the chest, which were subsequently reduced and brought down to the abdomen. Another mass was seen passing into this defect that was identified as the left kidney on palpation. This was also reduced and placed intra-abdominally. The posterior lip of the diaphragm was inadequate and medially unfurling was done, but there was gross deficiency. Therefore, the diaphragm was closed by suturing to the ribs and intercostal muscle using Prolene 2–0 in mattress sutures after mobilisation. The bowel was checked for malrotation. The duodenojejunal junction was to the left of the midline. Malrotation of the intestines was ruled out and the bowel was placed back intra-abdominally. After maintaining hemostasis, wounds were closed with interrupted layers using Vicryl.

**Figure 2:**
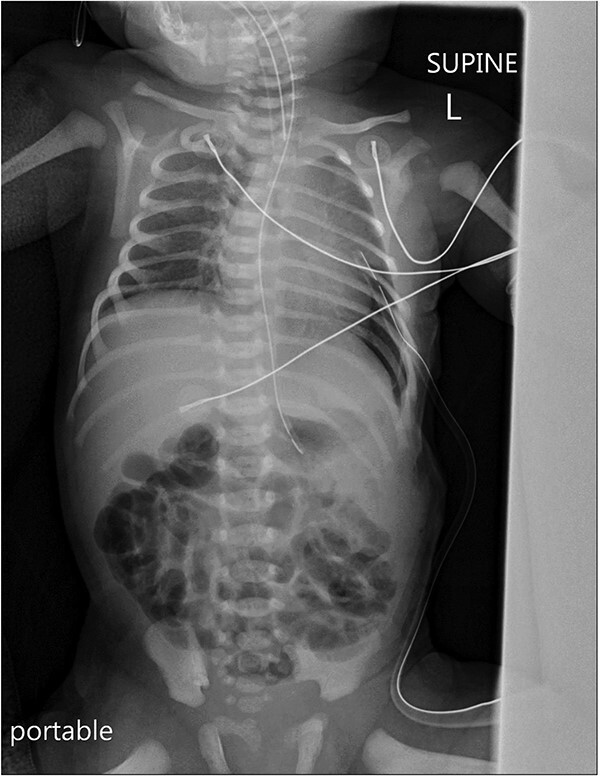
A portable X-ray photo of the chest, abdomen and pelvis taken postoperatively shows the expansion of both lungs and midline mediastinal position. A small subpulmonic pneumothorax on the left side is visible, for which a chest tube was inserted.

**Figure 3:**
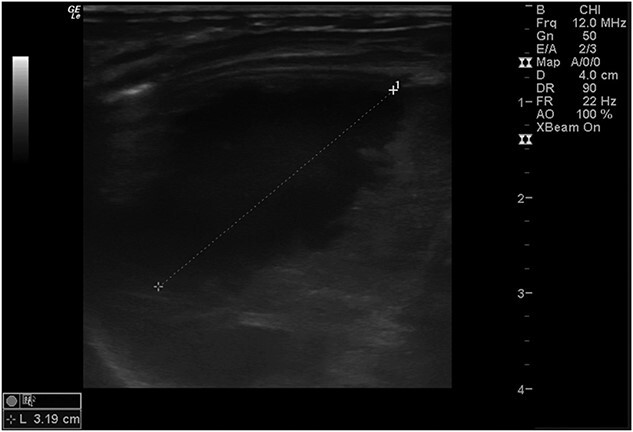
Abdominal ultrasound conducted 1 week post-operatively showed a possible splenic hematoma, which was self-limiting.

Postoperatively, a chest X-ray revealed a small left-sided pneumothorax that resolved in a few days (Fig. 2). Three days after the surgery, the fentanyl infusion was stopped, and the patient was extubated to a high-flow nasal cannula for 5 days. On the same day, the patient was noted to have a greenish-yellow aspirate. The patient was febrile the day after, a septic screen was done, and the patient was treated with ceftriaxone and vancomycin for 2 weeks. Furthermore, the surgical site was noted to have purulent discharge, for which fusidic acid was locally applied for 2 weeks. Abdominal ultrasound conducted a week after surgery showed an oval collection of fluid with intrinsic echoes, possibly a splenic hematoma, in the left upper quadrant, which was self-limited after 2 months of follow-up (Fig. 3). Before discharge, the patient’s parents were advised on a nursing plan, oral sucrose for analgesia and ranitidine.

## DISCUSSION

This case report described a patient diagnosed with a left-sided CDH shortly after birth and successfully treated surgically on his second day of life.

Global CDH prevalence is estimated to be 2.6 per 10 000 total births, with a mortality rate of 37.7%, the highest being in neonates aged 2–6 days old [2]. Current focuses of CDH management include supporting ventilation and preventing pulmonary hypertension and injury, achieving hemodynamic stability preoperatively, and prenatal screening and counselling [1, 4–7]. CDH is diagnosed by imaging, and there is an interest in improving prenatal diagnosis through ultrasound, which currently detects less than two-thirds of cases [8]. Therapy at this stage is experimental, but the most successful treatment has proven to be fetoscopic endoluminal tracheal occlusion between 27 and 29 weeks of gestation [8]. Postnatally, treatment emphasises stabilising the patient preoperatively using ventilation, managing pulmonary hypertension using inhaled nitric oxide as first-line treatment and hemodynamic support [8]. Corrective surgery is not emergent and it is not agreed upon when should it be performed, but the general consensus is that the patient must be physiologically stable [4, 8].

Future research must explore prenatal screening for congenital anomalies, including CDH, to allow for better treatment planning. There is an evident need for large-scale studies of CDH inclusive of diverse international populations [9]. Regional research should be updated, considering that the only study on CDH in an Emirati population was conducted in 1999 [10]. Since then, a literature search of CDH cases based in the UAE found one case treated with neurally adjusted ventilatory assist, one patient with Donnai–Barrow Syndrome from an inbred family and an Emirati family that had two pregnancies of three preterm-born children with Pallister–Killian syndrome who died within hours postnatally [11–13]. Finally, a thorough documentation of CDH cases is useful in retrospective studies [14].

## AUTHOR’S CONTRIBUTIONS

A.A. performed the surgery for this patient and contributed to the surgical treatment of the patient, obtained consent for this study and reviewed the final version of this paper. Z.A. and R.D. contributed to the manuscript by conducting and describing searches of the literature currently available and retrieving the records of the patient described in the case report. F.N. and F.E. contributed to the manuscript, study design, and supported the case description and discussion as well as reviewed the final version. T.A. reviewed the final version and contributed to the editing of the case report.

## CONFLICT OF INTEREST STATEMENT

None declared.

## FUNDING

No external funding was required for this study.

## ETHICAL APPROVAL

Study procedures were in accordance with the World Medical Association Declaration of Helsinki. Single case reports are exempted from ethical approval at our hospital.

## INFORMED CONSENT

Informed consent was obtained from the patient’s family.
